# Non-Canonical Notch Signaling Drives Activation and Differentiation of Peripheral CD4^+^ T Cells

**DOI:** 10.3389/fimmu.2014.00054

**Published:** 2014-02-12

**Authors:** Anushka Dongre, Lalitha Surampudi, Rebecca G. Lawlor, Abdul H. Fauq, Lucio Miele, Todd E. Golde, Lisa M. Minter, Barbara A. Osborne

**Affiliations:** ^1^Program in Molecular and Cellular Biology, University of Massachusetts Amherst, Amherst, MA, USA; ^2^Department of Veterinary and Animal Sciences, University of Massachusetts Amherst, Amherst, MA, USA; ^3^PAR, Chemical Synthesis Core Facility, Mayo Clinic Florida, Jacksonville, FL, USA; ^4^Cancer Institute, University of Mississippi Medical Center, Jackson, MS, USA; ^5^Department of Neuroscience, Center for Translational Research in Neurodegenerative Disease, McKnight Brain Institute, University of Florida, Gainesville, FL, USA

**Keywords:** Notch1, CD4^+^ T cell, non-canonical, activation, differentiation

## Abstract

Cleavage of the Notch receptor via a γ-secretase, results in the release of the active intra-cellular domain of Notch that migrates to the nucleus and interacts with RBP-Jκ, resulting in the activation of downstream target genes. This canonical Notch signaling pathway has been documented to influence T cell development and function. However, the mechanistic details underlying this process remain obscure. In addition to RBP-Jκ, the intra-cellular domain of Notch also interacts with other proteins in the cytoplasm and nucleus, giving rise to the possibility of an alternate, RBP-Jκ independent Notch pathway. However, the contribution of such RBP-Jκ independent, “non-canonical” Notch signaling in regulating peripheral T cell responses is unknown. In this report, we specifically demonstrate the requirement of Notch1 for regulating signal strength and signaling events distal to the T cell receptor in peripheral CD4^+^ T cells. By using mice with a conditional deletion in Notch1 or RBP-Jκ, we show that Notch1 regulates activation and proliferation of CD4^+^ T cells independently of RBP-Jκ. Furthermore, differentiation to T_H_1 and iTreg lineages although Notch dependent, is RBP-Jκ independent. Our striking observations demonstrate that many of the cell-intrinsic functions of Notch occur independently of RBP-Jκ. Such non-canonical regulation of these processes likely occurs through NF-κ B. This reveals a previously unknown, novel role of non-canonical Notch signaling in regulating peripheral T cell responses.

## Introduction

The Notch receptor protein plays a crucial role in embryonic development and specification of cell fates (1). There are four Notch receptors (Notch1–4), which can be activated by ligands that belong to either the Delta-like (DLL1, 3, 4) or Jagged family (Jagged 1 and 2) of proteins (2). Binding of a Notch ligand to the receptor triggers a series of proteolytic cleavages that culminate in the release of the intra-cellular domain of Notch by γ-secretase. This active, intra-cellular fragment of Notch migrates to the nucleus and interacts with the transcriptional repressor – recombination signal – binding protein-Jκ (RBP-Jκ). Following recruitment of co-activators such as p300 and master-mind-like (MAML), RBP-Jκ is converted to a transcriptional activator leading to the expression of downstream target genes. Such RBP-Jκ dependent or canonical Notch signaling has been long thought to regulate T cell responses. Recent reports suggest that the intra-cellular domain of Notch also interacts with other proteins besides RBP-Jκ in the cytoplasm and nucleus suggesting that Notch could possibly use an alternate route to exert some of its effects in an RBP-Jκ – independent or “non-canonical” fashion (3–6). However, whether or not such non-canonical Notch signaling is involved in regulating peripheral T cell responses is unknown.

T cell receptor (TCR) mediated activation of peripheral T cells is a fundamental process of the adaptive immune system. Activation of CD4^+^ T cells is accomplished by binding of an antigen to the TCR presented by an MHC Class II molecule on the antigen-presenting cell. A co-stimulatory signal between B7 (CD80/CD86) on the antigen-presenting cell and CD28 on the T cell stimulates the onset of multiple downstream signaling events, which result in T cell activation and proliferation. Helper T cells can differentiate into at least four different lineages (T_H_1, T_H_2, T_H_17, T-reg) depending on the cytokine milieu. Interleukin-12 and IFN-γ polarize CD4^+^ T cells to the T_H_1 phenotype. T_H_1 cells express the lineage specific transcription factor T-bet, secrete the signature cytokine IFN-γ, and provide protection against intra-cellular pathogens (7). T_H_2 cells, which are induced by IL-4, are primarily involved in asthma and allergies, and protect against extra-cellular parasites. They require the transcription factor GATA3 to secrete IL-4, IL-5, and IL-13 (8). Interleukin-6 and TGF-β generate T_H_17 cells, which provide protection against nematodes and fungal infections, secrete signature cytokines IL-17 and IL-23 and express ROR-γt (9–11). Induced regulatory T cells (iTregs), which are also induced by TGF-β, are characterized by the expression of FoxP3 and exhibit immuno-suppressive functions (12–14).

Ligation of the TCR accompanied by co-stimulation, generates intra-cellular Notch in CD4^+^ T cells while inhibition of Notch activation with γ-secretase inhibitors (GSI) decreases T cell activation as well as proliferation (15, 16). Since GSIs have multiple substrates and inhibit all Notch receptors (17), whether such a decrease in T cell activation is precisely due to Notch1, or a GSI induced effect, is ambiguous and needs to be investigated. Additionally, precisely the point in the TCR signaling cascade Notch at which Notch is involved remains to be determined. Several studies using various approaches to inhibit Notch activity have reported conflicting functions of Notch in regulating T cell activation as well as differentiation. Notch signaling has been shown to regulate differentiation to T_H_1, T_H_2, T_H_17, and iTreg lineages (4, 18–20). Pharmacological inhibition of Notch using GSIs dampens the ability to adopt a T_H_1 fate by attenuating the expression of T-bet (4). However, inhibition of signaling downstream of RBP-Jκ via genetic deletion or by using dominant negative MAML inhibits adoption of a T_H_2 fate *in vivo* while preserving a T_H_1 phenotype (21–23). Given the ability of intra-cellular Notch to interact with proteins different from RBP-Jκ, it is possible that disparate results could be attributed to RBP-Jκ independent functions of Notch. Furthermore, whether canonical and non-canonical Notch signaling affects T cell activation and differentiation processes differently requires further investigation.

In this study, we report that Notch is required for controlling signaling events distal to the T cell receptor and also acts as a critical regulator of TCR signal strength. We also show that activation and proliferation of peripheral CD4^+^ T cells specifically requires Notch1 but not RBP-Jκ since conditional deletion of Notch1 impaired these processes while conditional deletion of RBP-Jκ had no effect. Such non-canonical, RBP-Jκ independent regulation of these processes likely occurs via NF-κB. Conditional deletion of Notch1 also impaired polarization to T_H_1 and induction of regulatory T cells *in vitro*. Nevertheless, RBP-Jκ deficiency did not impair T_H_1 or iTreg cell fate *in vitro* once again supporting a novel role of non-canonical Notch signaling in controlling differentiation toward these lineages. *In vitro* polarization to T_H_2 was not affected in the absence of either Notch1 or RBP-Jκ. Our *in vitro* observations demonstrate a cell-intrinsic function of RBP-Jκ independent Notch signaling in regulating peripheral T cell responses. Such non-canonical regulation of these processes may serve to explain some of the differential, pleiotropic effects of Notch.

## Results

### Notch is required for distal TCR signaling events

**Figure 1:**
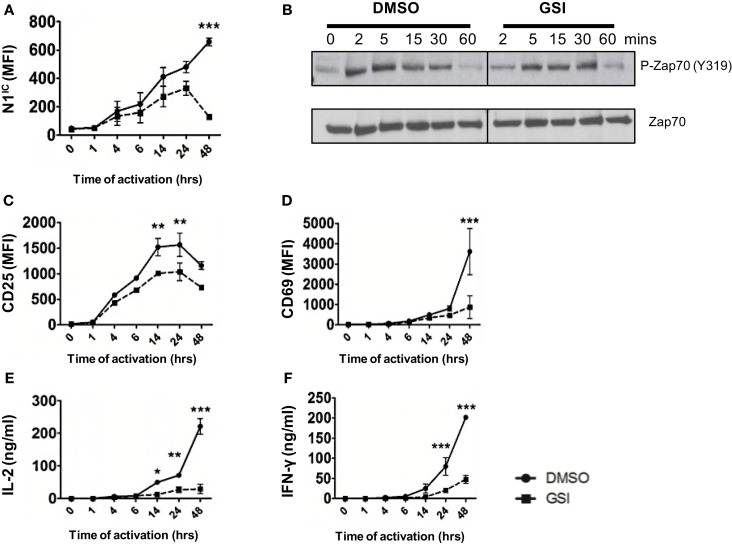
**Notch is required for distal TCR signaling events**. Splenocytes from C57BL/6J mice were pretreated with DMSO or GSI and stimulated with plate-bound anti-CD3ε and anti-CD28 for the indicated times. Cells were harvested and analyzed by flow cytometry after gating on CD4^+^ T cells. Mean fluorescent intensity (MFI) values were plotted for **(A)** N1^IC^, **(C)** CD25, and **(D)** CD69. **(E)** IL-2 and **(F)** IFN-γ ELISA from supernatants of cells stimulated as described. **(B)** Western Blot for phosphorylated and Total Zap70. Splenocytes from C57BL/6J mice were pretreated with DMSO or GSI and stimulated with anti-CD3ε and anti-CD28 for the indicated time points. Whole cell lysates were made at for each time point and analyzed by Western Blotting. Data are representative of three independent experiments. Data represent the mean ± SEM *n* = 3. *p < 0.05, **p < 0.005, and ***p < 0.001.

Activation of T cells via the TCR accompanied by co-stimulation leads to the production of the active, intra-cellular domain of Notch1 (N1^IC^) and its inhibition via γ-secretase inhibitors (GSI), decreases activation, and proliferation of T cells (15, 16). While Notch has been demonstrated to influence T cell activation, precisely where Notch exerts its influence downstream of the TCR is obscure. Furthermore, whether Notch affects signaling events proximal or distal to the TCR is unclear. To address these questions, we determined the kinetics of Notch activation over time and asked how inhibition of Notch activation via GSI treatment influences downstream TCR signaling events at early and late time points after stimulation. We detected N1^IC^ in CD4^+^ T cells activated with plate-bound anti-CD3ε and anti-CD28 4 h after activation and the amount of N1^IC^ increased over time (Figure 1A). This increase was abrogated after GSI treatment (Figure 1A). Inhibition of Notch activation did not alter proximal signaling events as evidenced by intact phosphorylation of Zap 70 even in GSI treated cells (Figure 1B). On the contrary, GSI treatment significantly decreased distal TCR signaling events such as the expression of activation markers CD25, CD69, IL-2, and IFN-γ (Figures 1C–F). This decrease was most prominent close to 48 h after TCR stimulation suggesting that Notch activation is critical for signaling events distal to the TCR, but could be dispensable for proximal events. Since we observed that activating cells via the TCR also triggered the activation of Notch, we determined whether CD4^+^ T cells themselves express Notch ligands. We observed that surface expression of DLL1 and Jagged1 is minimal upto 6 h after activation and peaks at distal time points (Figures S1A,B in Supplementary Material). Based on this observation, we determined whether stimulating T cells in the presence of recombinant Notch ligands alters the generation of N1^IC^ downstream of the TCR. Activation in the presence of recombinant DLL1 or Jagged1 did not alter the generation of N1^IC^ nor did it impact T cell activation (Figures S1C–H in Supplementary Material). Finally, stimulating T cells via the TCR in the presence of DLL1 or Jagged1 did not significantly influence the acquisition of helper T cell fate, although DLL1 enhanced IFN-γ production under pre-existing T_H_1 conditions (Figures S1I,K in Supplementary Material). Collectively, these data show that N1^IC^ is generated in CD4^+^ T cells after stimulating via the TCR and influences distal TCR signaling events.

### Notch as a regulator of signal strength

**Figure 2:**
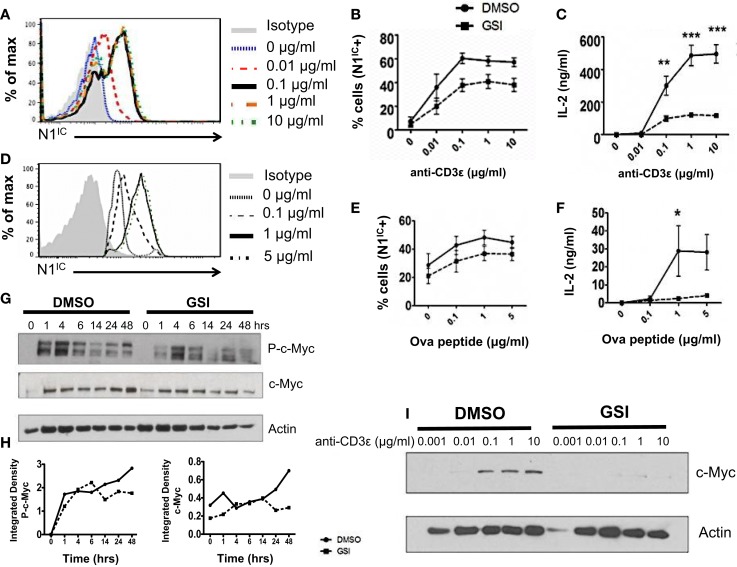
**Notch as a regulator of signal strength**. **(A)** Histogram for N1^IC^ expressed in CD4^+^ T cells stimulated with the indicated concentrations of anti-CD3ε and 1 μg/ml of anti-CD28 for 48 h. Data represent three independent experiments. **(B,C)** CD4^+^ T cells were pretreated with DMSO or GSI and activated with the indicated concentrations of anti-CD3ε and 1 μg/ml of anti-CD28 for 48 h. **(B)** Percentage of cells expressing N1^IC^ as determined by flow cytometry. **(C)** IL-2 ELISA from supernatants *n* = 3–5. **(D–F)** CD4^+^ T cells were isolated from DO.11.10 – TCR transgenic mice and pretreated with DMSO or GSI prior to co-culture with CHO-APCs expressing MHC-II and B7. They were pulsed with the indicated concentrations of ova-peptide 323–339. Cells were harvested after 48 h and analyzed by flow cytometry after gating on D0.11.10 TCR positive CD4^+^ T cells. **(D)** Histogram for N1^IC^ expressed in CD4^+^ T cells expressing the D0.11.10 TCR stimulated with the indicated concentrations of ova-peptide. Data represent three independent experiments. **(E)** Percentage of cells expressing N1^IC^ as determined by flow cytometry *n* = 3. **(F)** IL-2 ELISA from supernatants *n* = 3. **(G)** Western Blot for phosphorylated and total c-Myc. Splenocytes from C57BL/6J mice were pretreated with DMSO or GSI and stimulated with plate-bound anti-CD3ε and anti-CD28 for the indicated times. Whole cell lysates were made at each time and analyzed by Western Blotting. Data represent three independent experiments. **(H)** Integrated density values obtained after normalizing phospho and total c-Myc to Actin. **(I)** Western Blot for c-Myc expressed in CD4^+^ T cells stimulated as described in **(A)**. Data represent three independent experiments. Data represent the mean ± SEM, *p < 0.05, **p < 0.005, ***p < 0.001.

A possible role of Notch as a regulator of signal strength has been observed in thymocytes since constitutive expression of N1^IC^ in DP thymocytes prevented their maturation into single positive CD4^+^ and CD8^+^ T cells by interfering with TCR signal strength (24). However, whether Notch can influence the strength of TCR signaling in peripheral CD4^+^ T cells is unknown. Given the importance of Notch signaling in regulating activation, we asked whether it also influenced the threshold of signaling via the TCR. Since ligation of the TCR accompanied by co-stimulation was sufficient for the expression of N1^IC^, we first determined if altering signal strength could also influence the generation of N1^IC^. We stimulated CD4^+^ T cells with increasing concentrations of anti-CD3ε keeping the amount of anti-CD28 constant. Increasing signal strength resulted in a corresponding increase in the amount of N1^IC^ expression (Figure 2A). This was also accompanied by an increase in the percentage of cells expressing N1^IC^ and this increase could be inhibited by GSI treatment (Figure 2B). To determine if Notch is required for regulating signal strength, we stimulated cells with increasing amounts of anti-CD3ε after inhibiting Notch activation via GSI treatment. While DMSO treated cells secreted higher levels of IL-2 with increasing signal strength, GSI treated cells secreted significantly low amounts of IL-2 even at the highest signal strength of 10 μg/ml anti-CD3ε (Figure 2C) suggesting that Notch influenced the threshold of signaling via the TCR. To confirm this observation, we stimulated D0.11.10-TCR transgenic CD4^+^ T cells with antigen-presenting cells pulsed with increasing concentrations of ova-peptide. Once again, increasing antigen concentrations lead to a dose-dependent increase in the amount of N1^IC^ as well as the percentage of N1^IC^ positive cells (Figures 2D,E). This increase could be blocked after GSI treatment (Figure 2E). In addition to N1^IC^, increasing signal strength also led to an increase in IL-2. However in the absence of Notch activation, even high concentrations of ova-peptide were not sufficient for maximal IL-2 production (Figure 2F). These data demonstrate that not only can the levels of N1^IC^ be influenced by the amount of signal via the TCR, but also Notch itself can regulate signal strength by decreasing the threshold of signaling. In addition to Notch, the cell cycle regulator c-Myc is also influenced by TCR signal strength as strong peptide agonists cause a greater induction and nuclear translocation of c-Myc in T cells (25). c-Myc is a downstream target of Notch and is suspected to be important in thymocyte development and T cell function (25–28). Thus, we asked whether or not the regulation of c-Myc in response to signal strength was Notch dependent. We first confirmed Notch dependency of c-Myc in T cells. In concurrence with previous reports, we observed that phosphorylated and total c-Myc had a biphasic appearance in T cells and peaked at 4 and 24 h after TCR stimulation (28). However in addition to these results, we also observed that inhibition of Notch activation abrogated both phosphorylated and total c-Myc with the most prominent reduction at 48 h post stimulation (Figures 2G,H). Additionally, increasing signal strength lead to an increase in the expression of c-Myc, which was abrogated in the absence of Notch activation (Figure 2I). Thus, these data show that Notch is required for sustained c-Myc induction in peripheral CD4^+^ T cells. Furthermore, the induction of c-Myc in response to signal strength is Notch dependent.

### Notch1 is required for activation and proliferation of CD4^+^ T cells

**Figure 3:**
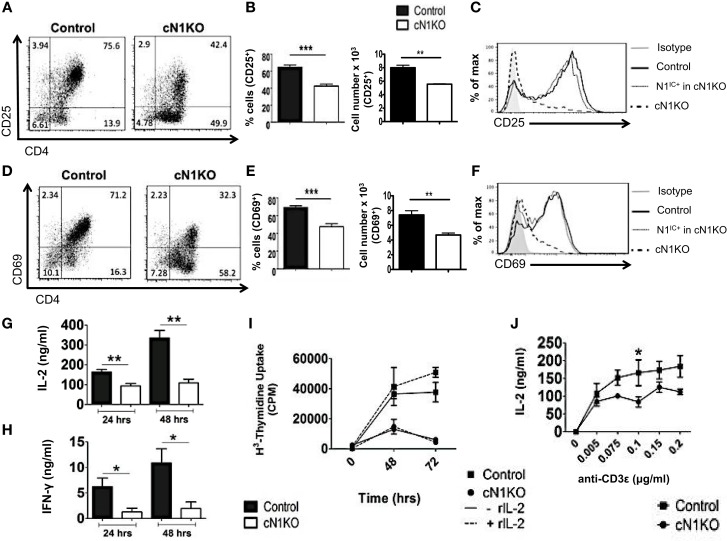
**Notch1 is required for activation and proliferation of CD4^+^ T cells**. CD4^+^ T cells were isolated from Control or cN1KO mice and stimulated with plate-bound anti-CD3ε and anti-CD28. Cells were harvested after 48 h **(A–F,J)** or at indicated times **(G–I)**. Cells were surface stained for CD4, CD25, and CD69 and intra-cellular stained for N1^IC^ and analyzed by flow cytometry. **(A,D)** Dot plots obtained after flow cytometry. Data are representative of four independent experiments. Numbers in each quadrant represent percentage of cells. **(B)** Percentage of cells and cell counts for cells positive for CD25 and **(E)** CD69. **(A,D)** *n* = 4. **(C,F)** Histograms for CD25 and CD69 after gating on Notch negative cells from cN1KO mice (dashed line), Notch positive cells from cN1KO mice (dotted line), or CD4^+^ T cells from Control mice (solid line). Data represent four independent experiments. **(G)** IL-2 and **(H)** IFN-γ ELISA from supernatants obtained from control and cN1KO mice stimulated as described above *n* = 4. **(I)** Counts per minute (CPM) obtained after ^3^H-thymidine uptake in CD4^+^ T cells from control and cN1KO mice stimulated as described above with and without rmIL-2 (20 ng/ml). Data represent three independent experiments. **(J)** IL-2 ELISA from supernatants of CD4^+^ T cells from Control or cN1KO mice stimulated with the indicated concentrations of anti-CD3ε and 1 μg/ml of anti-CD28 for 48 h *n* = 3–5. Data represent mean ± SEM. *p < 0.05, **p < 0.005.

Inhibition of Notch activation using GSIs has been demonstrated to abrogate activation and proliferation of CD4^+^ T cells (15, 16). However, GSIs inhibit all isoforms of Notch and have multiple substrates (17). Hence, the specific role of Notch1 in controlling these processes requires further investigation. To determine the specific function of Notch1 in T cell activation and differentiation, we conditionally knocked-out Notch1 in peripheral T cells by crossing mice with loxp-flanked Notch1 alleles, to mice expressing Cre recombinase under the control of the interferon responsive Mx promoter (29). The Mx Cre promoter enables acute deletion of Notch1 in peripheral T cells. Since Notch1 is required for development of T cells (30–32), this deletion strategy enables cells to develop normally in the presence of Notch, before its deletion in the periphery. Both Notch1*^fl^*^/^*^fl^* Mx Cre^±^mice (abbreviated as cN1KO) and Notch1*^fl^*^/^*^fl^* Mx Cre^−/−^mice (abbreviated as control) were injected with equivalent amounts of Poly I:Poly C and rested for 3 weeks before use (29). CD4^+^ T cells from cN1KO animals showed a significant decrease in Notch1 transcript as well as a marked reduction in the amount of N1^IC^ expression (Figures S2A,D in Supplementary Material) (29). We also confirmed the expression of other Notch receptors in CD4^+^ T cells from control and cN1KO animals and observed an increase in the expression of Notch2 and Notch3 transcripts in the absence of Notch1 (Figures S2B,C in Supplementary Material). We could not detect Notch4 in cells from either control or cN1KO mice. In addition, both cN1KO and control mice expressed similar percentages of CD4^+^ and CD8^+^ peripheral T cells (Figures S2E,F in Supplementary Material). To investigate the specific contribution of Notch1 in influencing activation of peripheral T cells, CD4^+^ T cells from cN1KO or control animals were stimulated *in vitro* with anti-CD3ε and anti-CD28 and activation markers were observed by flow cytometry. cN1KO animals had a significantly lower percentage as well as absolute number of cells expressing CD25 and CD69 (Figures 3A,B,D,E). Since cN1KO mice were knocked-down for Notch1, as an internal control we gated on the small percentage of residual Notch1 positive cells in the cN1KO animals. These cells continued to express high levels of CD25 and CD69. However, cells deficient for Notch1 expressed lower levels of both activation markers (Figures 3C,F). This decrease was accompanied by a significant reduction in the levels of IL-2 and IFN-γ secreted post-activation (Figures 3G,H). Whether CD4^+^ T cells from cN1KO animals were also impaired in their ability to proliferate was determined by measuring the incorporation of ^3^H-thymidine. Proliferative capability was significantly diminished in the absence of Notch1 and could not be rescued by adding exogenous IL-2 (Figure 3I). To determine if the observed decrease in proliferation was due to enhanced apoptosis in the absence of Notch1, Annexin V positive cells from control or cN1KO animals were analyzed by flow cytometry. CD4^+^ T cells from cN1KO animals had only marginally more apoptosis 48 h after activation (Figures S3A,B in Supplementary Material). Since Notch1 was required for the activation and proliferation of CD4^+^ T cells, we asked whether it also influenced the threshold of signaling via the TCR. CD4^+^ cells from control and cN1KO animals were stimulated with increasing concentrations of anti-CD3ε accompanied by co-stimulation. While cells from control mice secreted higher levels of IL-2 with increasing signal strength, cells from cN1KO mice had low levels of IL-2 even at the highest signal strength of 0.2 μg/ml anti-CD3ε (Figure 3J). These results demonstrate that activation and proliferation of CD4^+^ T cells specifically requires Notch1. Furthermore, Notch1 also acts to decrease the threshold for signaling via the TCR.

### Notch1 is required for T_H_1 differentiation and production of iTregs *in vitro*

**Figure 4:**
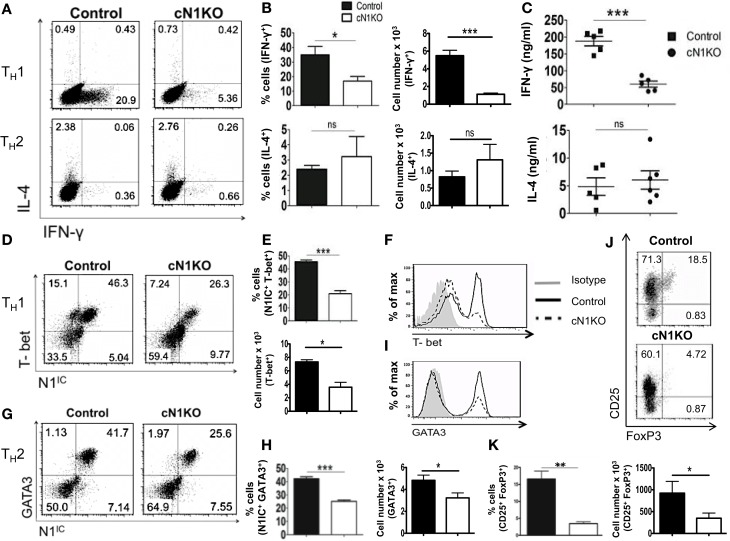
**Notch1 is required for T_H_1 differentiation and production of iTregs *in vitro***. CD4^+^ T cells from control and cN1KO mice were differentiated under T_H_1, T_H_2, or iTreg inducing conditions for 3 days followed by re-stimulation with plate-bound anti-CD3ε. Cells were analyzed by flow cytometry. Supernatants were used for ELISA. **(A)** Dot plots obtained from flow cytometry showing intra-cellular staining for IFN-γ and IL-4. Numbers in each quadrant represent percentage of cells. Data represent three to five independent experiments. **(B)** Percentage and absolute cell numbers of IFN-γ or IL-4 positive cells determined by flow cytometry *n* = 3–5. **(C)** IFN-γ and IL-4 production under T_H_1 and T_H_2 conditions, respectively, determined by an ELISA. Each data point represents one animal. **(D,G)** Dot plots showing intra-cellular staining for **(D)** T-bet and N1^IC^ under T_H_1 conditions **(G)** or GATA3 and N1^IC^ under T_H_2 conditions. Numbers in each quadrant represent percentage of cells. Data represent three to five independent experiments. **(E,H)** Percentage and absolute numbers of double positive cells as determined by flow cytometry *n* = 3–5. **(F)** Histograms for T-bet and **(I)** GATA3 expression under T_H_1 and T_H_2 conditions, respectively. Data represent three to five independent experiments. **(J)** Dot plots for CD25^+^ and FoxP3^+^ cells. Numbers in each quadrant represent percentage of cells. Data represent three independent experiments. **(K)** Percentage and absolute cell numbers of double positive cells determined by flow cytometry *n* = 3. Data represent mean ± SEM. *p < 0.05, **p < 0.005, and ***p < 0.001. ns, not significant.

Although Notch has been implicated in influencing differentiation of T cells, the precise role of Notch1 in favoring T_H_1 versus T_H_2 lineage decisions is unclear. While some studies have shown that inhibiting Notch using GSIs diminishes the ability of CD4^+^ T cells to adopt a T_H_1 fate, other studies using different strategies to inhibit Notch signaling have reported conflicting observations (21, 22, 33). To determine the precise role of Notch1 in helper T cell differentiation, CD4^+^ T cells from control or cN1KO mice were polarized *in vitro* to T_H_1, T_H_2, or iTreg lineages. Absence of Notch1 impaired T_H_1 differentiation *in vitro*. CD4^+^ T cells from cN1KO mice had significantly fewer cells that stained positive for intra-cellular IFN-γ (Figures 4A,B). Secreted IFN-γ was also reduced significantly in the absence of Notch1 (Figure 4C). This decrease was accompanied by a reduction in the amount of the master T_H_1 transcription factor T-bet (Figures 4D–F). In contrast, no marked effect was observed in T_H_2 differentiation. Although, the amount of GATA3 was reduced in the absence of Notch1 (Figures 4G–I), both control as well as cN1KO mice had similar percentages and absolute number of CD4^+^ T cells that were positive for intra-cellular IL-4 (Figures 4A,B) and expressed comparable levels of secreted IL-4 (Figure 4C). Whether CD4^+^ T cells from cN1KO mice proliferated differently under different polarizing conditions, was determined by ^3^H-thymidine uptake under non-polarized (NP), T_H_1 and T_H_2 conditions. Proliferative capability of CD4^+^ T cells from cN1KO mice was the same under different polarizing conditions (Figure S3C in Supplementary Material), despite of differences in cytokine secretion. In addition to T_H_1, Notch1 deficiency significantly reduced induced T-reg populations as observed by a significant decrease in the frequency of CD25^+^FoxP3^+^ cells in cN1KO animals (Figures 4J,K). These results show that Notch1 is required for T_H_1 and iTreg differentiation but is dispensable for T_H_2 cell fate acquisition *in vitro*. Furthermore, these data demonstrate an intrinsic role of Notch1 in regulating helper T cell differentiation.

### Canonical Notch signaling is not required for activation and proliferation of CD4^+^ T cells

**Figure 5:**
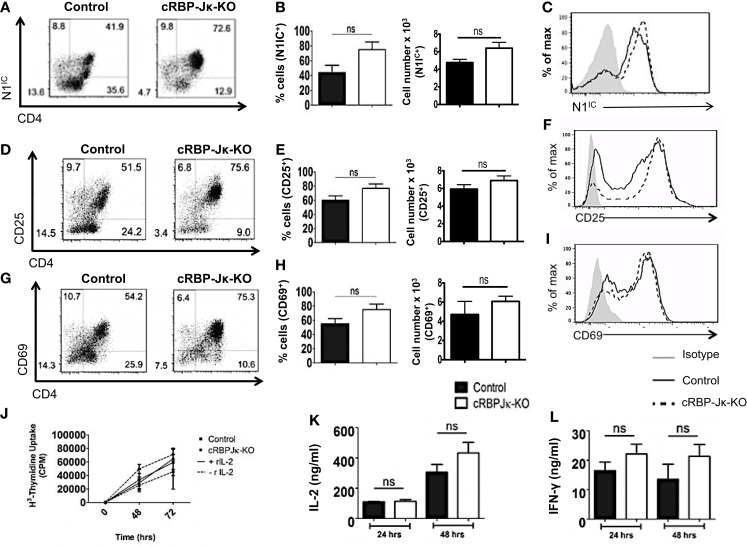
**Canonical Notch signaling is not required for activation and proliferation of CD4^+^ T cells**. CD4^+^ T cells were isolated from control or cRBP-Jκ-KO mice and stimulated with plate-bound anti-CD3ε and anti-CD28. Cells were harvested after 48 h **(A–I)** or at indicated times. **(J–L)** Cells were surface stained for CD4, CD25, CD69, and intra-cellular stained for N1^IC^ and analyzed by flow cytometry. **(A,D,G)** Dot plots obtained from flow cytometry showing CD4^+^ T cells positive for **(A)** N1^IC^, **(D)** CD25, and **(G)** CD69. Numbers in each quadrant represent percentage of cells. Data represent three independent experiments. **(B,E,H)** Percentage and absolute numbers of cells positive for **(B)** N1^IC^, **(E)** CD25, and **(H)** CD69 obtained from dot plots *n* = 4. **(C,F,I)** Histograms for **(C)** N1^IC^, **(F)** CD25, and **(I)** CD69. Data represent three independent experiments. **(J)** Counts per minute (CPM) obtained after ^3^H-thymidine uptake in CD4^+^ T cells from control and cRBP-Jκ-KO mice stimulated as described above with and without rmIL-2 (20 ng/ml). Data represent three independent experiments. **(K)** IL-2 and **(L)** IFN-γ ELISA from supernatants obtained from control and cRBP-Jκ-KO mice stimulated as described above *n* = 6. Data represent mean ± SEM, ns, not significant.

Several studies have highlighted an emerging role of non-canonical Notch signaling in controlling helper T cell differentiation (5, 34). This is potentiated by interactions of the intra-cellular domain of Notch with other proteins besides RBP-Jκ in the cytoplasm and nucleus. Some of these alternate binding partners include (but are not limited to) NF-κB, T-bet, GATA3, PI3K, and Akt (3–5, 15, 21–23, 35). However, whether canonical and non-canonical Notch signaling differs in their ability to influence T cell activation and differentiation is not well defined. Hence, to determine the importance of RBP-Jκ-dependent, canonical Notch signaling, we conditionally knocked-out RBP-Jκ in peripheral T cells by breeding mice carrying RBP-Jκ loxp-flanked sites to mice expressing Cre recombinase under the control of the Mx promoter. CD4^+^ T cells from *RBP-J*κ*^fl^*^/^*^fl^* Mx Cre^±^ mice (abbreviated as cRBP-Jκ-KO) expressed substantially reduced transcript and protein levels of RBP-Jκ in comparison to to CD4^+^ T cells from *RBP-J*κ*^fl^*^/^*^fl^* Mx Cre^−/−^ mice (abbreviated as controls) (Figures S4A,B in Supplementary Material). Additionally, cRBP-Jκ-KO mice had significantly fewer peripheral CD4^+^ and CD8^+^ T cells in their spleens (Figures S4C,D in Supplementary Material). To determine how canonical Notch signaling influenced activation, CD4^+^ T cells from control and cRBP-Jκ-KO mice were stimulated *in vitro* with anti-CD3ε and anti-CD28. Absence of RBP-Jκ did not alter the production of intra-cellular Notch1 after TCR stimulation (Figures 5A–C). In contrast to impaired activation observed in the absence of Notch1 (Figure 3), RBP-Jκ deficiency did not alter the activation of CD4^+^ T cells. On the contrary, CD4^+^ T cells from cRBP-Jκ-KO animals produced marginally higher numbers of CD4^+^ T cells expressing activation the markers CD25 and CD69 and displayed slightly elevated amounts of each marker (Figures 5D–I). CD4^+^ T cells from both cRBP-Jκ-KO mice secreted IL-2 and IFN-γ just as efficiently as Controls (Figures 5K,L). Proliferation of CD4^+^ T cells was unaffected by the absence of RBP-Jκ irrespective of addition of recombinant IL-2 (Figure 5J). These results suggest that activation and proliferation of CD4^+^ T cells is not impaired in the absence of RBP-Jκ. However, since these processes required Notch1, our data show that activation and proliferation occurs independently of canonical Notch signaling.

### Activation and proliferation of CD4^+^ T cells is RBP-Jκ-independent but Notch and NF-κB dependent

**Figure 6:**
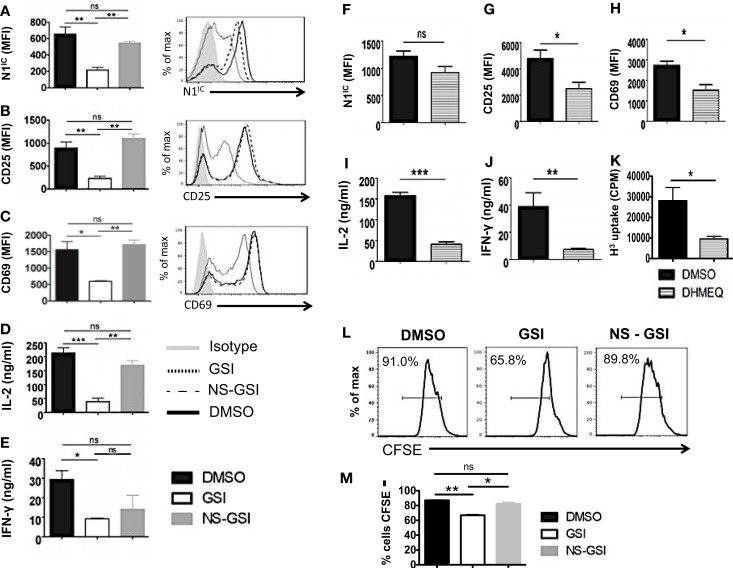
**Activation and Proliferation of CD4^+^ T cells is RBP-Jκ independent but Notch and NF-κB dependent**. CD4^+^ T cells were isolated from cRBP-Jκ-KO mice, pretreated with DMSO, GSI, or NS-GSI **(A–E,L,M)** or DHMEQ **(F–J)** and stimulated with plate-bound anti-CD3ε and anti-CD28 for 24 h. Cells were surface stained for CD4, CD25, and CD69 and intra-cellular stained for N1^IC^ and analyzed by flow cytometry. Supernatants were used to detect IL-2 and IFN-γ by an ELISA mean fluorescent intensity (MFI) values were plotted for **(A,F)** N1^IC^, **(B,G)** CD25, **(C,H)** CD69 *n* = 3–5. Histograms to the right of **(A–C)** show expression of N1^IC^, CD25, and CD69. Data represent three independent experiments. **(D,I)** IL-2 and **(E,J)** IFN-γ as determined by an ELISA. **(K)** Counts per minute (CPM) obtained after ^3^H-thymidine uptake in CD4^+^ T cells from cRBP-Jκ-KO mice treated with DMSO or DHMEQ and stimulated for 48 h. **(L,M)** Histograms representing a CFSE Proliferation Assay. CD4^+^ T cells from cRBP-Jκ-KO mice pretreated with DMSO, GSI, or NS-GSI were labeled with CFSE and activated with plate-bound anti-CD3ε and anti-CD28 for 48 h followed by flow cytometry analysis. Data represent three independent experiments. **(M)** Bar graph showing the percentage of CFSE negative cells obtained by flow cytometry. Data represent three independent experiments. Data represent mean ± SEM. *p < 0.05, **p < 0.005, and ***p < 0.001. ns, not significant.

To confirm that RBP-Jκ-independent activation and proliferation was in fact Notch dependent but RBP-Jκ-independent, we used the following strategies. We first inhibited activation of Notch in CD4^+^ T cells from cRBP-Jκ-KO by treating these cells with GSI. To control for the off-target effects of GSIs, we also treated cells with a Notch sparing GSI (NS-GSI) that inhibited all GSI substrates except Notch. GSI treatment of CD4^+^ T cells from cRBP-Jκ-KO mice inhibited intra-cellular Notch (Figure 6A) and significantly reduced the expression of the activation markers CD25 and CD69 (Figures 6B,C). This was accompanied by a significant decrease in the cytokines IL-2 and IFN-γ (Figures 6D,E). Importantly, NS-GSI treatment rescued Notch activation as well as CD25, CD69, and IL-2 (Figures 6A–D). A partial rescue was observed with IFN-γ (Figure 6E). Furthermore, a decrease in proliferation of CD4^+^ T cells from cRBP-Jκ-KO mice after GSI treatment was also rescued by the NS-GSI (Figures 6L,M). These data suggest that while canonical Notch signaling is dispensable for the activation and proliferation of peripheral CD4^+^ T cells, these processes require intra-cellular Notch. The role of Notch and NF-κB in regulating T cell activation and differentiation processes has been well documented (3, 15, 36, 37). We have shown that the intra-cellular domain of Notch1 binds to and exerts some of its effects via NF-κB suggestive of cross talk between the two pathways (3, 15). Hence, we asked whether these RBP-Jκ-independent processes, were also dependent on NF-κB. This was determined by examining activation markers after inhibiting NF-κB in CD4^+^ T cells lacking RBP-Jκ using a pharmacological inhibitor, dehydroxymethylepoxyquinomicin (DHMEQ) (38). DHMEQ has been used to block NF-κB activity in different types of solid tumors, malignant cells, and T cells (39, 40). DHMEQ treatment effectively inhibited nuclear translocation of NF-κB (Figures S6A,B in Supplementary Material). Although DHMEQ treatment did not alter the levels of N1^IC^ (Figure 6F), DHMEQ treated CD4^+^ T cells from cRBP-Jκ-KO mice showed a significant reduction in the amounts of CD25, CD69, IL-2, and IFN-γ (Figures 6G–J). Furthermore, DHMEQ treatment of CD4^+^ T cells from cRBP-Jκ-KO animals significantly impaired proliferation (Figure 6K). These data show that activation and proliferation of CD4^+^ T cells is an RBP-Jκ-independent but Notch dependent process. Furthermore, our data suggest that non-canonical Notch signaling may control these processes, at least in part through NF-κB.

### RBP-Jκ-deficiency does not alter CD4^+^ T cell differentiation *in vitro*

**Figure 7:**
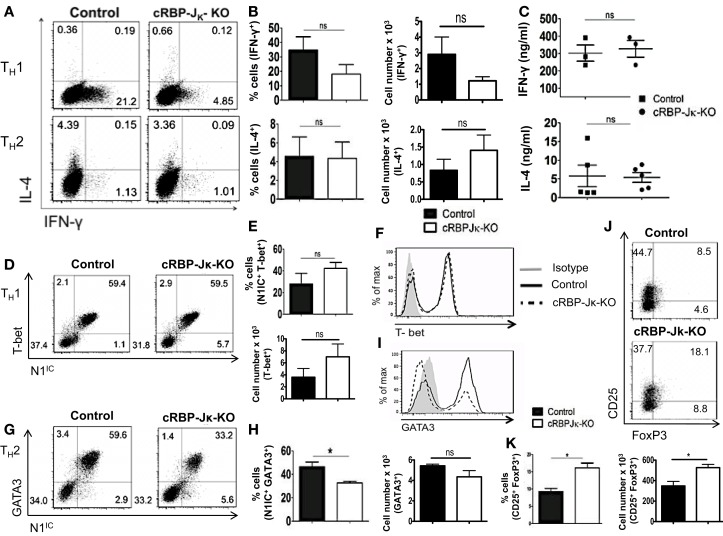
**RBP-Jκ deficiency does not alter CD4^+^ T cell differentiation *in vitro***. CD4^+^ T cells from control and cRBP-Jκ-KO mice were differentiated under T_H_1, T_H_2, or iTreg inducing condition for 3 days followed by re-stimulation with plate-bound anti-CD3. Cells were analyzed by flow cytometry. Supernatants were used for ELISA. **(A)** Dot plots showing intra-cellular staining for IFN-γ and IL-4. **(B)** Percentage and absolute numbers of IFN-γ or IL-4 positive cells determined by flow cytometry. Numbers in each quadrant represent percentage of cells *n* > 3. **(C)** IFN-γ and IL-4 production under T_H_1 and T_H_2 conditions respectively determined by an ELISA. Each data point represents one animal. **(D,G)** Dot plots showing intra-cellular staining for **(D)** T-bet and N1^IC^ under T_H_1 conditions and **(G)** GATA3 and N1^IC^ under T_H_2 conditions. **(E,H)** Percentages and absolute numbers of double positive cells determined by flow cytometry *n* = 5. **(F,I)** Histograms for T-bet and GATA3 expression under T_H_1 and T_H_2 conditions, respectively. Data represent at least three independent experiments. **(J)** Flow cytometry plots for CD25^+^ and FoxP3^+^ cells. **(K)** Percentages and absolute numbers of double positive cells determined by flow cytometry *n* = 3. Data represent mean ± SEM. *p < 0.05, ns, not significant. Data represent three to five independent experiments.

Deletion of either Notch1 or RBP-Jκ has been shown to have different outcomes on helper T cell differentiation, suggesting that acquisition of helper T cell fate may be differentially influenced by canonical and non-canonical Notch signaling. Whether the absence of RBP-Jκ influenced polarization of CD4^+^ T cells *in vitro* was determined by skewing cells from control or cRBP-Jκ-KO animals toward T_H_1 and T_H_2 cell fates. While the number of cells secreting IFN-γ was reduced in the CD4^+^ T cells from cRBP-Jκ-KO mice (Figures 7A,B), the amount of IFN-γ secreted under T_H_1 conditions was unaffected (Figure 7C). Levels of T-bet remained unchanged in the absence of RBP-Jκ in comparison to controls (Figures 7D–F). Similarly, RBP-Jκ-deficiency did not alter polarization toward T_H_2. Although, CD4^+^ T cells from RBP-Jκ-KO animals had lower amounts of GATA3 (Figures 7G–I) this decrease did not influence the number of IL-4 positive cells (Figures 7A,B) or the amount of secreted IL-4 under T_H_2 conditions *in vitro* (Figure 7C). In addition to T_H_1 and T_H_2 phenotypes, the absence of RBP-Jκ did not impair the ability to induce regulatory T cells. The number of CD25^+^Foxp3^+^ cells was significantly increased in cRBP-Jκ-KO animals (Figures 7J,K). These data show that differentiation of CD4^+^ T cells *in vitro* does not require canonical Notch signaling.

## Discussion

Several studies have demonstrated the generation of the active, intra-cellular domain of Notch in T cells stimulated via the TCR accompanied by co-stimulation (15, 16). In this report, we address precisely where Notch exerts influence downstream of the TCR signaling cascade. We specifically outline the kinetics of Notch activation in peripheral T cells and suggest that Notch is most important for regulating signaling events distal to the TCR. We show that inhibition of Notch activation had no effect on phosphorylation of Zap70, a proximal TCR signaling event. However, GSI treatment effectively abolished the expression of T cell activation markers – CD25, CD69, IL-2, and IFN-γ, events that occur several hours following TCR stimulation. In addition, GSI treatment also abolished the expression of c-Myc most prominently at 14–48 h post TCR stimulation. While these data do not exclude a role of Notch in affecting other early TCR events besides phosphorylation of Zap70, they suggest that Notch has a very significant influence on distal events. Furthermore, our data also reveal a critical influence of Notch activation on TCR signal strength. We show that stimulating T cells with increasing concentrations of either anti-CD3ε or antigen pulsed APCs, increased the amount of N1^IC^ in proportion to increasing signal strength. Although, we did observe basal levels of N1^IC^ expression in CD4^+^ T cells stimulated with CHO-APCs in the absence of ova-peptide, CHO-APCs express low levels of Jagged 1 and likely contribute to the basal expression of N1^IC^. Most importantly, abrogating Notch activation either via GSI treatment or conditional deletion significantly increased the threshold of signaling via the TCR. While Notch has been implicated in influencing strength of signal in thymocytes (23), our data demonstrate for the first time a role of Notch as a regulator of signal strength in peripheral CD4^+^ T cells. We also observed a concomitant increase in c-Myc in response to increasing signal. While a similar response has been recently documented in peripheral T cells (24), our data add to these data by showing that an increase in c-Myc in response to increasing signal strength is, in fact, Notch dependent. These data also suggest that Notch may likely exert control over signal strength via c-Myc. However, further experimentation is required to investigate the precise mechanism that underlies Notch dependent regulation of signal strength.

Many studies have implicated a role of Notch in regulating peripheral T cell responses using GSIs to inhibit Notch activity (4, 15, 16). However, the use of GSIs obscures the specific contribution of Notch1 in regulating these processes since GSIs inhibit all isoforms of the Notch receptor and have multiple substrates, some of which are important in immune function (17). Thus, GSI treatment may not only inhibit Notch but other type I transmembrane proteins as well suggesting that attenuation of T cell responses post GSI treatment may not necessarily be due to Notch1. Here, we specifically address such concerns by conditionally deleting Notch1 using the Mx Cre system, which produces “acute” deletion of Notch1 in peripheral T cells. CD4^+^ T cells from cN1KO animals showed a significant reduction in CD25, CD69, IL-2, and IFN-γ coupled with impaired proliferation that could not be rescued in the presence of exogenous IL-2. In addition, although CD4^+^ T cells from cN1KO animals expressed Notch2 and Notch3, it was not sufficient to rescue activation or proliferation. Thus, our data show that activation and proliferation are in fact Notch dependent processes that specifically require Notch1.

The precise function of Notch signaling in determining T_H_1 versus T_H_2 lineage decisions remains controversial partially due to the disparate methods used to attenuate Notch signaling. Inhibition of Notch signaling via GSIs blocked T_H_1 cell fate *in vitro* and *in vivo* by preventing Notch mediated up-regulation of T-bet but did not alter T_H_2 responses *in vitro* (4). On the contrary, inhibition of RBP-Jκ function either by genetic deletion or by using dominant negative co-activators of RBP-Jκ such as dnMAML (dominant negative MAML), which mimics a loss-of-Notch function phenotype, preserved T_H_1 responses but dampened T_H_2 responses *in vivo* (18, 21–23, 33). In models of RBP-Jκ deletion, the generation of N1^IC^ is preserved (Figure 5). This is particularly important since N1^IC^ has also been documented to interact with other proteins besides RBP-Jκ (3–5). Therefore, we reasoned that since Notch1 is known to interact with proteins other than RBP-Jκ, N1^IC^ may be capable of functioning in an RBP-Jκ independent fashion and such “non-canonical” signaling could serve to reconcile existing differences about the precise role of Notch in influencing T cell differentiation. To this end, we generated mice with a conditional deletion of either Notch1 or RBP-Jκ and determined whether deleting different components of the Notch pathway produced distinct phenotypes. Furthermore, we specifically chose to study how the absence of Notch signaling affects T cell differentiation *in vitro* to delineate a cell-intrinsic role of Notch in controlling effector T cell responses in contrast to previously used *in vivo* approaches, which cannot distinguish between extrinsic and intrinsic effects.

We show that conditionally deleting Notch1 attenuates T_H_1 responses *in vitro* as observed by a significant decrease in the percentage of cells secreting IFN-γ, the amount of secreted IFN-γ and the amount of T-bet expressed suggesting that Notch1 is in fact required for T_H_1 decisions. Another study has shown that deleting Notch1 under the control of a CD4 Cre promoter does not dampen T_H_1 responses *in vitro*. We suggest that the differences between this study and ours may most likely be due to the different approaches used to delete Notch since deletion under the control of the CD4 Cre promoter, deletes Notch during thymic development. Apart from T_H_1, we also show that deletion of Notch1 impaired the ability to generate induced regulatory T cells. Inhibition of Notch via GSIs has also been shown to decrease iTreg populations, suggestive of a requirement for Notch1 in regulating these responses. Although, we observed a decrease in both T_H_1 and iTreg populations in the absence of Notch1, we did not see any decrease in T_H_2 responses *in vitro*. We have shown previously that inhibition of Notch via GSIs under T_H_2 conditions does not alter IL-4 production (4). Corroborating these observations, another study showed that genetic deletion of presenilin, a component of the γ-secretase complex, did not alter T_H_2 responses *in vitro* (41). Our T_H_2 data concur with these reports suggesting that Notch1 is dispensable for intrinsic acquisition of a T_H_2 cell fate *in vitro*.

To determine the contribution of RBP-Jκ dependent, canonical Notch signaling in regulating activation and differentiation of peripheral CD4^+^ T cells, we conditionally deleted RBP-Jκ once again under the control of the Mx promoter facilitating acute deletion. CD4^+^ T cells from cRBP-Jκ-KO animals expressed N1^IC^ upon TCR stimulation. Strikingly, contrary to CD4^+^ T cells from cN1KO animals, CD4^+^ T cells from cRBP-Jκ-KO animals were not deficient in activation or proliferation and expressed all activation markers at identical levels to CD4^+^ T cells from control mice suggesting an RBP-Jκ independent role of Notch signaling in regulating these processes. We confirmed this by showing that only after intra-cellular Notch is inhibited in CD4^+^ T cells lacking RBP-Jκ, activation and proliferation can be decreased. Additionally, an NS-GSI could “rescue” activation and proliferation in the absence of RBP-Jκ. The NS-GSI only partially rescued IFN-γ. A possible explanation for this observation is that Notch may regulate IFN-γ production via an intermediary molecule, which is a GSI target and is hence not spared by the NS-GSI. While these data suggest only a partial requirement of N1^IC^ for IFN-γ production, determining the precise molecular players that interact with Notch to regulate IFN-γ, requires further experimentation. Our data concur with previously reported observations that showed no overt effect of RBP-Jκ deletion on T cell activation or proliferation (23, 41–43). However, our study is the first to suggest that this is due to non-canonical Notch signaling as N1^IC^ could compensate for the absence of RBP-Jκ.

The mechanism by which non-canonical Notch signaling regulates activation, proliferation, and differentiation requires further investigation. Our data along with that of others suggest NF-κB to be the most likely candidate (3, 15, 36, 37). Thus, to determine if some of the effects of non-canonical Notch signaling require NF-κB, we inhibited NF-κB in the absence of RBP-Jκ. Inhibition of NF-κB did not alter the levels of intra-cellular Notch1 but still decreased the expression of CD25, CD69, IL-2, and IFN-γ in CD4^+^ cells from cRBP-Jκ-KO animals NF-κB inhibition also attenuated proliferation in the absence of RBP-Jκ. Furthermore, attenuation of NF-κB in the absence of RBP-Jκ attenuated a T_H_1 response *in vitro* since DHMEQ treatment reduced the expression of IFN-γ and T-bet in cells lacking RBP-Jκ (Figures S6C,D in Supplementary Material), suggesting that RBP-Jκ independent but Notch1 dependent regulation of these responses may require NF-κB. Additionally, inhibiting NF-κB significantly affected T cell activation only at a distal time point of 48 h and abolished the expression of c-Myc suggesting that Notch may require NF-κB to control these processes (Figure S5 in Supplementary Material). However, deciphering the precise mechanism of such NF-κB mediated non-canonical Notch signaling is an ongoing area of investigation that requires further experimentation.

To extend the contribution of non-canonical Notch signaling to helper T cell differentiation, we validated T_H_1, T_H_2, and iTreg responses *in vitro* in the absence of RBP-Jκ. In stark contrast to CD4^+^ T cells from cN1KO animals which showed a markedly dampened T_H_1 response, CD4^+^ T cells from cRBP-Jκ-KO mice secreted IFN-γ and expressed T-bet just as efficiently as controls. These data once again showcase RBP-Jκ independent, but Notch1 dependent regulation of effector T cell responses *in vitro*. A recent study has also suggested such RBP-Jκ independent Notch signaling in regulating T_H_1 responses *in vivo* by showing that CD4^+^ T cells lacking RBP-Jκ could mount a protective response to *Leishmania major* but those lacking both the Notch1 and Notch2 receptors could not (34). In addition to T_H_1, we observed an increase in the number of CD25^+^FoxP3^+^ double positive cells in the absence of RBP-Jκ suggesting that induction of regulatory T cells may also rely on non-canonical Notch signaling. This observation was in contrast to a significant decrease of the same subset seen in the absence of Notch1 (Figures 4J,K). These differences can be reconciled by the fact that cRBP-Jκ-KO mice continue to express elevated levels of N1^IC^. Since N1^IC^ is required for T-reg lineage determination, an increase in N1^IC^ expression in the absence of RBP-Jk, could be responsible for a significant increase in the regulatory T cell population. Additionally, since RBP-Jκ is a transcriptional repressor, deletion of RBP-Jκ could de-repress FoxP3, resulting in an increase in the number of CD25^+^FoxP3^+^ T-regs. Finally, we did not observe an effect of RBP-Jκ deficiency on the secretion of T_H_2 cytokines *in vitro*. Our data do not support an intrinsic role for Notch1 signaling in T_H_2 responses. However, since Notch1 acts upstream of IL-4 and Notch1 has been shown to regulate IL-4 secretion by NKT cells, we suggest that Notch1 regulates T_H_2 responses extrinsically and may instead regulate *in vivo* IL-4 production.

In conclusion, our *in vitro* approach resolves discrepancies about the role of Notch signaling in CD4^+^ T cell function by showing that Notch1 regulates T cell activation, proliferation, and differentiation in a cell-intrinsic fashion. Importantly, our data demonstrate for the first time that RBP-Jκ independent, non-canonical Notch signaling regulates activation, proliferation, and acquisition of T_H_1 and iTreg fates *in vitro*. Such non-canonical Notch signaling most likely involves NF-κB. Evidence of non-canonical Notch signaling has been observed during axon guidance as well as dorsal closure during embryonic development in *Drosophila* (44, 45). In addition, a cytosolic function of Notch is required for survival of neural stem cells (46). Non-canonical Notch signaling has recently been shown to influence the IL-6/JAK/STAT pathway in breast tumors in a fashion that requires NF-κB (47). Additionally, mammary tumor development has been shown to occur independently of RBP-Jκ. Further studies are required to delineate the precise molecular mechanisms underlying non-canonical Notch signaling however our data as well as others support a role for NF-κB in mediating non-canonical Notch signaling.

## Materials and Methods

### Animals

All animals were housed in animal facilities as per the guidelines approved by the Institutional Animal Care and Use Committee at the University of Massachusetts-Amherst. C57BL/6J mice and BALB/c-Tg (DO11.10)10Loh/J were purchased from the Jackson Laboratory (Bar Harbor, ME, USA). cN1KO and cRBP-Jκ-KO mice were generated by breeding *Notch1^fl^*^/^*^fl^* (*Notch1^tm2Rko/GridJ^*) or *Rbp-j*κ*^fl^*^/^*^fl^* (*Rbpj^tm1Hon^*) mice to *mx1Cre*^±^ [B6.Cg-Tg(mx1cre)1Cgn/J] mice from the Jackson Laboratory (Bar Harbor, ME, USA). All mice – *Notch1^fl^*^/^*^fl^* × Mx Cre^±^ mice (cN1KO), *Notch1^fl^*^/^*^fl^* × Mx Cre^−/−^ (Control), *RBP-J*κ*^fl/fl^* × Mx Cre^±^(cRBP-Jκ-KO) and *RBP-J*κ*^fl^*^/^*^fl^* × Mx Cre^−/−^ (Control) were injected with 12–15 μg/g body weight of *Poly I:Poly C* (GE Healthcare, Imgenex) every other day for 5 days. Animals were sacrificed after a 3-week resting period. Mice aged 7–12 weeks were used for all experiments.

### T cell isolation and *in vitro* assays

CD4^+^ T cells were isolated by magnetic separation using anti-CD4 magnetic particles (BD Pharmingen). Cells were activated *in vitro* by plating 3 × 10^6^ cells/ml on each well of a 12-well plate pre-coated with anti-CD3ε and anti-CD28 purified from 145-2c11 and 37N hybridoma cell lines respectively and cross-linked with anti-Hamster IgG (Sigma). Cells were activated in a half and half mixture of RPMI and DMEM (Lonza) supplemented with 10% Fetal Bovine Serum (GIBCO), l-Glutamine, Na-pyruvate, and Penicillin/Streptomycin (Lonza). The following conditions were used for polarization. T_H_1: 10 μg/ml anti-IL-4 and 1 ng/ml recombinant mouse IL-12 (BD Pharmingen). T_H_2: 10 μg/ml anti-IFN-γ and 1 ng/ml of recombinant mouse IL-4 (BD Pharmingen). iTreg: CD4^+^CD25^−^ cells were enriched from bulk splenocytes using the mouse CD4 T-lymphocyte enrichment set with 2.5 μg biotin conjugated anti-CD25 (BD Pharmingen). Cells were activated in the presence of 2 ng/ml of recombinant human TGFβ1 (R&D systems). Cytokines from supernatants were determined using a standard ELISA assay (BD Pharmingen). For thymidine uptake assays, 3 × 10^5^ cells/ml were activated in 96-well plates pre-coated with anti-CD3ε and anti-CD28 in the presence or absence of 20 ng/ml recombinant mouse IL-2 (BD Pharmingen). Cells were pulsed with ^3^H-thymidine (Perkin Elmer) at a final concentration of 1 μci/rxn in the final 16 h of activation. Cells were harvested on a mash harvester into scintillation vials. Counts per minute were obtained on a scintillation counter (Beckman). For CFSE assays, CD4^+^ T cells were labeled with 1 μM CFSE using the manufacturer’s protocol (Molecular Probes). Cells were stimulated for 48 h before analysis by flow cytometry. For drug treatments, CD4^+^ T cells were pretreated with 50 μM of the GSI z-IL-CHO, 5 μM NS-GSI JLK-6 (Tocris Bioscience), or 2 μM of DHMEQ at 37°C for 30 min before activation.

### Strength of signal assays

CD4^+^ T cells were isolated from the spleens of C57BL/6J as described and stimulated with the indicated concentrations of anti-CD3ε and 1 μg/ml of anti-CD28 (BD Pharmingen) for 48 h. Cells were analyzed by flow cytometry and IL-2 was detected in the supernatants by an ELISA. For co-culture experiments, empty vector Chinese Hamster Ovary – Antigen-presenting cells (CHO-APCs) were fixed by treating with Mitomycin C (Sigma) 37°C for 45 min 6 × 10^5^ cells/ml of CHO-APCs were pulsed with the indicated concentrations of ova-peptide 323–339 (Genscript). CD4^+^ T cells were isolated from spleens of BALB/c-Tg(DO11.10)10Loh/J and mixed with peptide pulsed APCs at 2.5 × 10^6^ cells/ml in each well of a 12-well plate. Cells were harvested after 48 h and analyzed by flow cytometry after gating on CD4^+^ T cells expressing the DO.11.10-TCR. Supernatants were used for detection of IL-2 by an ELISA.

### Flow cytometry

Surface staining was performed in PBS with 1% BSA using the indicated antibodies – CD25-APC, CD69-FITC (eBioscience), CD4-PerCP (BD Pharmingen), Anti-mouse DO.11.10-TCR Biotin (Invitrogen), Streptavidin PerCP (BD Pharmingen). Intra-cellular staining was performed for detection of intra-cellular Notch1, T-bet, GATA3, and FoxP3 using the FoxP3 staining buffer set (eBioscience) and the following antibodies: anti-Human/Mouse Notch1-PE, anti-Human/Mouse T-bet PE-Cy7, anti-Human/Mouse GATA3 eFluor 660, anti-Mouse/Rat FoxP3 Alexa 488 (eBioscience). For detection of intra-cellular cytokines, cells were harvested at indicated time points and re-stimulated with plate-bound anti-CD3ε in the presence of Golgi Plug (IFN-γ) or Golgi Stop (IL-4) (BD Pharmingen) for 5 h. Intra-cellular cytokine staining was performed using the BD Cytofix/CytoPerm plus kit and cytokines detected using anti-Mouse IFN-γ FITC and anti-Mouse IL-4 PE (BD Pharmingen). Flow cytometry data was acquired on a FACS LSR II (BD) and analyzed using FlowJo software (Trestar) after gating on CD4^+^ T cells or as indicated.

### Western blot

Whole cell lysates were made in RIPA buffer (150 mM NaCl, 1% IgeCal-CA 360, 0.1% SDS, 50 mM Tris, pH-8.0, 0.5% Sodium deoxycholate). Cytoplasmic and nuclear proteins were extracted as per the manufacturer’s instructions (Thermo Scientific). Lysates were resolved on an SDS-PAGE gel. Protein was a transferred on a nitro-cellulose membrane and blocked in Blotto (5% milk powder, 0.2% Tween-20 in PBS). Membranes were probed over-night with primary antibody. Membranes were washed and incubated with horse-radish peroxidase (HRP) labeled secondary antibody. Membranes were developed using ECL reagents (Amersham). Primary Antibodies: anti-RBPSUH, anti-phospho Zap 70 (Y319), anti-Zap70, anti-HDAC (Cell signaling), anti-c-Rel (Santa Cruz Biotechnology) anti-Actin (Sigma), anti-c-Myc (9E10) was obtained from Dr. Dominique Alfandari. Secondary Antibody: anti-Rabbit-HRP, anti-Mouse-HRP (Amersham).

### Statistical analysis

All data are represented as mean ± SEM. Statistical Analysis was performed using the GraphPad Prism 5 software. *p* Values were calculated using an unpaired two-tailed Student’s *t*-test.

## Author Contributions

Anushka Dongre, Lalitha Surampudi, Rebecca G. Lawlor performed experiments and analyzed data. Anushka Dongre, Lalitha Surampudi, Rebecca G. Lawlor, and Barbara A. Osborne designed experiments with contributions from Lisa M. Minter, Lucio Miele, and Todd E. Golde. Abdul H. Fauq synthesized GSIs and DHMEQ. Barbara A. Osborne conceived the study, supervised experimental design and interpretation of data. Anushka Dongre and Barbara A. Osborne wrote the manuscript.

## Conflict of Interest Statement

The authors declare that the research was conducted in the absence of any commercial or financial relationships that could be construed as a potential conflict of interest.

## Supplementary Material

The Supplementary Material for this article can be found online at http://www.frontiersin.org/Journal/10.3389/fimmu.2014.00054/abstract

## References

[1] Artavanis-Tsakonas S Rand MD Lake RJ Notch signaling: cell fate control and signal integration in development. Science (1999) 284(5415):770–6 10.1126/science.284.5415.770 10221902

[2] Maillard I Fang T Pear WS Regulation of lymphoid development, differentiation, and function by the Notch pathway. Annu Rev Immunol (2005) 23:945–74 10.1146/annurev.immunol.23.021704.115747 15771590

[3] Shin HM Minter LM Cho OH Gottipati S Fauq AH Golde TE Notch1 augments NF-kappaB activity by facilitating its nuclear retention. EMBO J (2006) 25(1):129–38 10.1038/sj.emboj.7600902 16319921 PMC1356346

[4] Minter LM Turley DM Das P Shin HM Joshi I Lawlor RG Inhibitors of gamma-secretase block in vivo and in vitro T helper type 1 polarization by preventing Notch upregulation of Tbx21. Nat Immunol (2005) 6(7):680–8 10.1038/ni1209 15991363

[5] Perumalsamy LR Nagala M Banerjee P Sarin A A hierarchical cascade activated by non-canonical Notch signaling and the mTOR-Rictor complex regulates neglect-induced death in mammalian cells. Cell Death Differ (2009) 16(6):879–89 10.1038/cdd.2009.20 19265851

[6] Minter LM Osborne BA Canonical and non-canonical Notch signaling in CD4(+) T cells. Curr Top Microbiol Immunol (2012) 360:99–114 10.1007/82_2012_233 22695917

[7] Szabo SJ Sullivan BM Stemmann C Satoskar AR Sleckman BP Glimcher LH Distinct effects of T-bet in TH1 lineage commitment and IFN-gamma production in CD4 and CD8 T cells. Science (2002) 295(5553):338–42 10.1126/science.1065543 11786644

[8] Zheng W Flavell RA The transcription factor GATA-3 is necessary and sufficient for Th2 cytokine gene expression in CD4 T cells. Cell (1997) 89(4):587–96 10.1016/S0092-8674(00)80240-8 9160750

[9] Yang XO Pappu BP Nurieva R Akimzhanov A Kang HS Chung Y T helper 17 lineage differentiation is programmed by orphan nuclear receptors ROR alpha and ROR gamma. Immunity (2008) 28(1):29–39 10.1016/j.immuni.2007.11.016 18164222 PMC2587175

[10] Murphy CA Langrish CL Chen Y Blumenschein W McClanahan T Kastelein RA Divergent pro- and antiinflammatory roles for IL-23 and IL-12 in joint autoimmune inflammation. J Exp Med (2003) 198(12):1951–7 10.1084/jem.20030896 14662908 PMC2194162

[11] Langrish CL Chen Y Blumenschein WM Mattson J Basham B Sedgwick JD IL-23 drives a pathogenic T cell population that induces autoimmune inflammation. J Exp Med (2005) 201(2):233–40 10.1084/jem.20041257 15657292 PMC2212798

[12] Chen W Jin W Hardegen N Lei KJ Li L Marinos N Conversion of peripheral CD4+CD25- naive T cells to CD4+CD25+ regulatory T cells by TGF-beta induction of transcription factor Foxp3. J Exp Med (2003) 198(12):1875–86 10.1084/jem.20030152 14676299 PMC2194145

[13] Fu S Zhang N Yopp AC Chen D Mao M Zhang H TGF-beta induces Foxp3 + T-regulatory cells from CD4 + CD25 – precursors. Am J Transplant (2004) 4(10):1614–27 10.1111/j.1600-6143.2004.00566.x 15367216

[14] Rao PE Petrone AL Ponath PD Differentiation and expansion of T cells with regulatory function from human peripheral lymphocytes by stimulation in the presence of TGF-{beta}. J Immunol (2005) 174(3):1446–55 15661903 10.4049/jimmunol.174.3.1446

[15] Palaga T Miele L Golde TE Osborne BA TCR-mediated Notch signaling regulates proliferation and IFN-gamma production in peripheral T cells. J Immunol (2003) 171(6):3019–24 12960327 10.4049/jimmunol.171.6.3019

[16] Adler SH Chiffoleau E Xu L Dalton NM Burg JM Wells AD Notch signaling augments T cell responsiveness by enhancing CD25 expression. J Immunol (2003) 171(6):2896–903 12960312 10.4049/jimmunol.171.6.2896

[17] Beel AJ Sanders CR Substrate specificity of gamma-secretase and other intramembrane proteases. Cell Mol Life Sci (2008) 65(9):1311–34 10.1007/s00018-008-7462-2 18239854 PMC2569971

[18] Amsen D Blander JM Lee GR Tanigaki K Honjo T Flavell RA Instruction of distinct CD4 T helper cell fates by different Notch ligands on antigen-presenting cells. Cell (2004) 117(4):515–26 10.1016/S0092-8674(04)00451-9 15137944

[19] Keerthivasan S Suleiman R Lawlor R Roderick J Bates T Minter L Notch signaling regulates mouse and human Th17 differentiation. J Immunol (2011) 187(2):692–701 10.4049/jimmunol.1003658 21685328 PMC3131467

[20] Samon JB Champhekar A Minter LM Telfer JC Miele L Fauq A Notch1 and TGFbeta1 cooperatively regulate Foxp3 expression and the maintenance of peripheral regulatory T cells. Blood (2008) 112(5):1813–21 10.1182/blood-2008-03-144980 18550850 PMC2518888

[21] Amsen D Antov A Jankovic D Sher A Radtke F Souabni A Direct regulation of Gata3 expression determines the T helper differentiation potential of Notch. Immunity (2007) 27(1):89–99 10.1016/j.immuni.2007.05.021 17658279 PMC2062505

[22] Fang TC Yashiro-Ohtani Y Del Bianco C Knoblock DM Blacklow SC Pear WS Notch directly regulates Gata3 expression during T helper 2 cell differentiation. Immunity (2007) 27(1):100–10 10.1016/j.immuni.2007.04.018 17658278 PMC2801546

[23] Tu L Fang TC Artis D Shestova O Pross SE Maillard I Notch signaling is an important regulator of type 2 immunity. J Exp Med (2005) 202(8):1037–42 10.1084/jem.20050923 16230473 PMC2213210

[24] Izon DJ Punt JA Xu L Karnell FG Allman D Myung PS Notch1 regulates maturation of CD4+ and CD8+ thymocytes by modulating TCR signal strength. Immunity (2001) 14(3):253–64 10.1016/S1074-7613(01)00107-8 11290335

[25] Guy CS Vignali KM Temirov J Bettini ML Overacre AE Smeltzer M Distinct TCR signaling pathways drive proliferation and cytokine production in T cells. Nat Immunol (2013) 14(3):262–70 10.1038/ni.2538 23377202 PMC3577985

[26] Douglas NC Jacobs H Bothwell AL Hayday AC Defining the specific physiological requirements for c-Myc in T cell development. Nat Immunol (2001) 2(4):307–15 10.1038/86308 11276201

[27] Lindsten T June CH Thompson CB Multiple mechanisms regulate c-myc gene expression during normal T cell activation. EMBO J (1988) 7(9):2787–94 3053165 10.1002/j.1460-2075.1988.tb03133.x PMC457069

[28] Nie Z Hu G Wei G Cui K Yamane A Resch W c-Myc is a universal amplifier of expressed genes in lymphocytes and embryonic stem cells. Cell (2012) 151(1):68–79 10.1016/j.cell.2012.08.033 23021216 PMC3471363

[29] Roderick JE Gonzalez-Perez G Kuksin CA Dongre A Roberts ER Srinivasan J Therapeutic targeting of NOTCH signaling ameliorates immune-mediated bone marrow failure of aplastic anemia. J Exp Med (2013) 210(7):1311–29 10.1084/jem.20112615 23733784 PMC3698520

[30] Radtke F Wilson A Stark G Bauer M van Meerwijk J MacDonald HR Deficient T cell fate specification in mice with an induced inactivation of Notch1. Immunity (1999) 10(5):547–58 10.1016/S1074-7613(00)80054-0 10367900

[31] Deftos ML Huang E Ojala EW Forbush KA Bevan MJ Notch1 signaling promotes the maturation of CD4 and CD8 SP thymocytes. Immunity (2000) 13(1):73–84 10.1016/S1074-7613(00)00009-1 10933396 PMC2780426

[32] Wolfer A Bakker T Wilson A Nicolas M Ioannidis V Littman DR Inactivation of Notch 1 in immature thymocytes does not perturb CD4 or CD8T cell development. Nat Immunol (2001) 2(3):235–41 10.1038/85294 11224523

[33] Tanigaki K Tsuji M Yamamoto N Han H Tsukada J Inoue H Regulation of alphabeta/gammadelta T cell lineage commitment and peripheral T cell responses by Notch/RBP-J signaling. Immunity (2004) 20(5):611–22 10.1016/S1074-7613(04)00109-8 15142529

[34] Auderset F Schuster S Coutaz M Koch U Desgranges F Merck E Redundant Notch1 and Notch2 signaling is necessary for IFNgamma secretion by T helper 1 cells during infection with *Leishmania major*. PLoS Pathog (2012) 8(3):e1002560 10.1371/journal.ppat.1002560 22396647 PMC3291656

[35] Sade H Krishna S Sarin A The anti-apoptotic effect of Notch-1 requires p56lck-dependent, Akt/PKB-mediated signaling in T cells. J Biol Chem (2004) 279(4):2937–44 10.1074/jbc.M309924200 14583609

[36] Barbarulo A Grazioli P Campese AF Bellavia D Di Mario G Pelullo M Notch3 and canonical NF-kappaB signaling pathways cooperatively regulate Foxp3 transcription. J Immunol (2011) 186(11):6199–206 10.4049/jimmunol.1002136 21508258

[37] Vacca A Felli MP Palermo R Di Mario G Calce A Di Giovine M Notch3 and pre-TCR interaction unveils distinct NF-kappaB pathways in T-cell development and leukemia. EMBO J (2006) 25(5):1000–8 10.1038/sj.emboj.7600996 16498412 PMC1409728

[38] Ueki S Yamashita K Aoyagi T Haga S Suzuki T Itoh T Control of allograft rejection by applying a novel nuclear factor-kappaB inhibitor, dehydroxymethylepoxyquinomicin. Transplantation (2006) 82(12):1720–7 10.1097/01.tp.0000250548.13063.44 17198266

[39] Nishioka C Ikezoe T Jing Y Umezawa K Yokoyama A DHMEQ, a novel nuclear factor-kappaB inhibitor, induces selective depletion of alloreactive or phytohaemagglutinin-stimulated peripheral blood mononuclear cells, decreases production of T helper type 1 cytokines, and blocks maturation of dendritic cells. Immunology (2008) 124(2):198–205 10.1111/j.1365-2567.2007.02755.x 18217958 PMC2566624

[40] Horie R Watanabe M Okamura T Taira M Shoda M Motoji T DHMEQ, a new NF-kappaB inhibitor, induces apoptosis and enhances fludarabine effects on chronic lymphocytic leukemia cells. Leukemia (2006) 20(5):800–6 10.1038/sj.leu.2404167 16525497

[41] Ong CT Sedy JR Murphy KM Kopan R Notch and presenilin regulate cellular expansion and cytokine secretion but cannot instruct Th1/Th2 fate acquisition. PLoS One (2008) 3(7):e2823 10.1371/journal.pone.0002823 18665263 PMC2474705

[42] Kopan R Ilagan MX The canonical Notch signaling pathway: unfolding the activation mechanism. Cell (2009) 137(2):216–33 10.1016/j.cell.2009.03.045 19379690 PMC2827930

[43] Tanigaki K Honjo T Regulation of lymphocyte development by Notch signaling. Nat Immunol (2007) 8(5):451–6 10.1038/ni1453 17440450

[44] Crowner D Le Gall M Gates MA Giniger E Notch steers *Drosophila* ISNb motor axons by regulating the Abl signaling pathway. Curr Biol (2003) 13(11):967–72 10.1016/S0960-9822(03)00325-7 12781136

[45] Zecchini V Brennan K Martinez-Arias A An activity of Notch regulates JNK signalling and affects dorsal closure in *Drosophila*. Curr Biol (1999) 9(9):460–9 10.1016/S0960-9822(99)80211-5 10322111

[46] Androutsellis-Theotokis A Leker RR Soldner F Hoeppner DJ Ravin R Poser SW Notch signalling regulates stem cell numbers in vitro and in vivo. Nature (2006) 442(7104):823–6 10.1038/nature04940 16799564

[47] Jin S Mutvei AP Chivukula IV Andersson ER Ramskold D Sandberg R Non-canonical Notch signaling activates IL-6/JAK/STAT signaling in breast tumor cells and is controlled by p53 and IKKalpha/IKKbeta. Oncogene (2013) 32(41):4892–902 10.1038/onc.2012.517 23178494 PMC3795477

