# Supplementary figures and images for: Non-Canonical Notch Signaling Drives Activation and Differentiation of Peripheral CD4+ T Cells
Source: Front Immunol. 2014 Feb 12;5:54. doi: 10.3389/fimmu.2014.00054 (PMC3921607; doi:10.3389/fimmu.2014.00054)

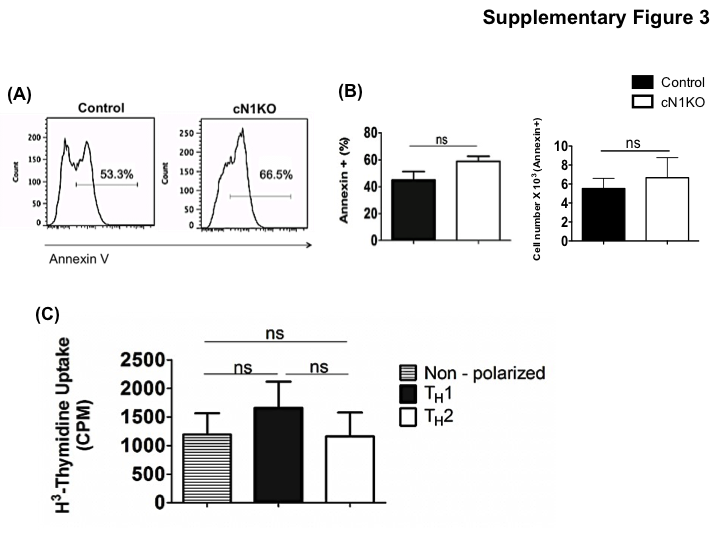

Supplement: Supplementary file 1 [file 76414_Osborne_Presentation1.ZIP › Supple/76414_Osborne_Figure_10.TIF]

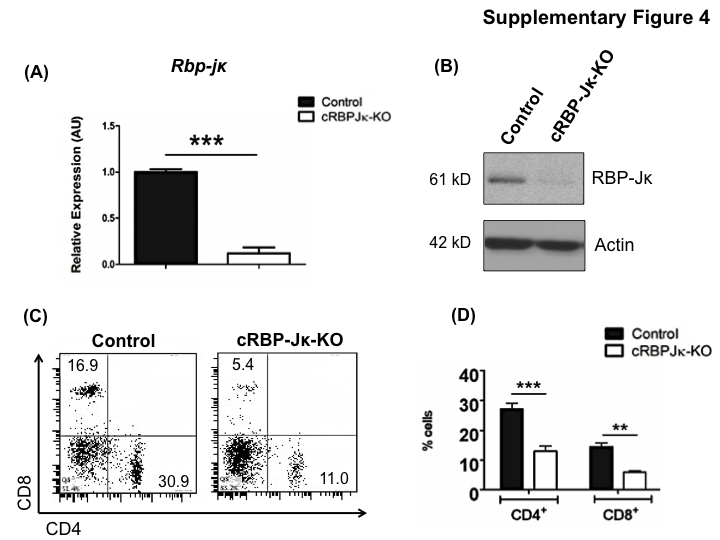

Supplement: Supplementary file 1 [file 76414_Osborne_Presentation1.ZIP › Supple/76414_Osborne_Figure_11.TIF]

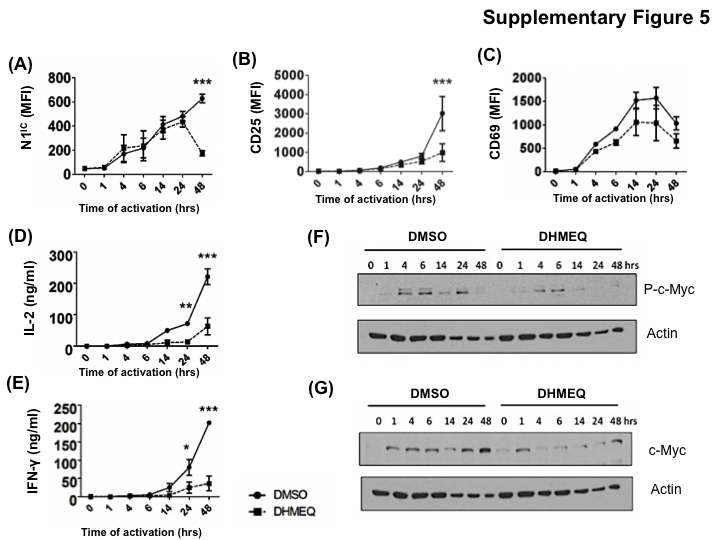

Supplement: Supplementary file 1 [file 76414_Osborne_Presentation1.ZIP › Supple/76414_Osborne_Figure_12.TIF]

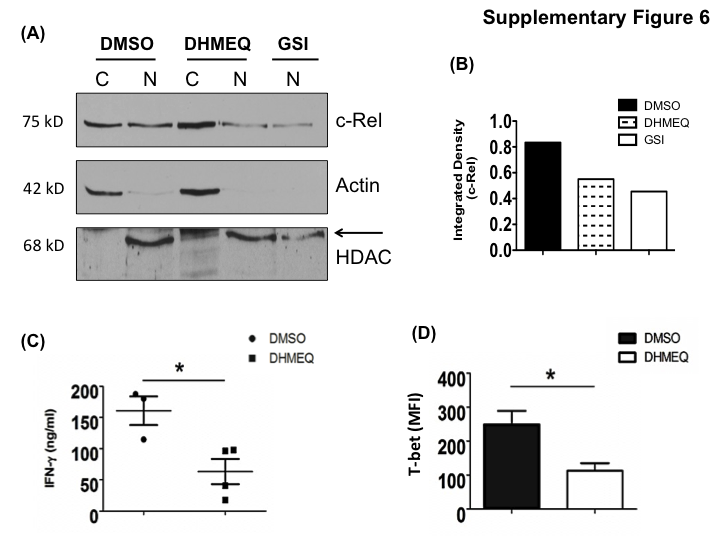

Supplement: Supplementary file 1 [file 76414_Osborne_Presentation1.ZIP › Supple/76414_Osborne_Figure_13.TIF]

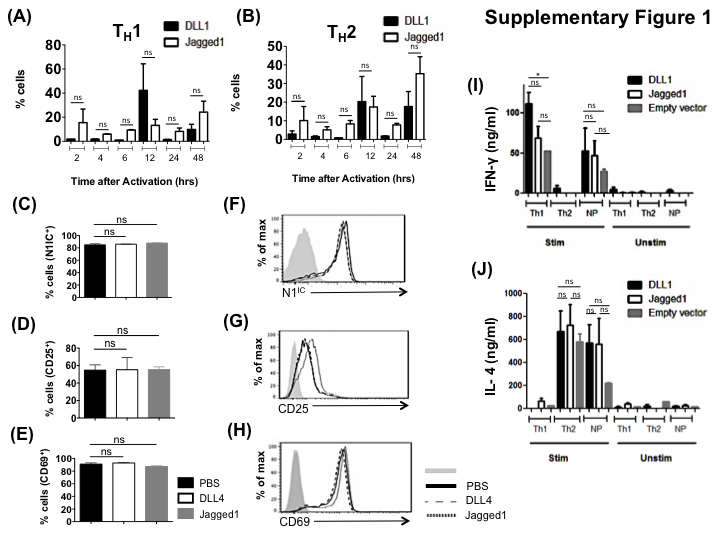

Supplement: Supplementary file 1 [file 76414_Osborne_Presentation1.ZIP › Supple/76414_Osborne_Figure_8.TIF]

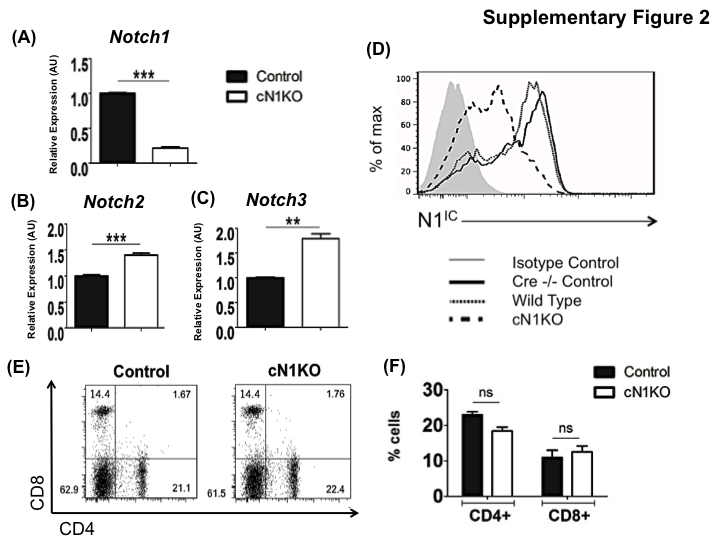

Supplement: Supplementary file 1 [file 76414_Osborne_Presentation1.ZIP › Supple/76414_Osborne_Figure_9.TIF]
